# Nomogram prediction for central lymph node metastasis in papillary thyroid microcarcinoma of the isthmus based on clinical and ultrasound features

**DOI:** 10.3389/fsurg.2026.1728250

**Published:** 2026-01-22

**Authors:** Yunbin Shi, Lihui Qian, Juntao Huang, Tao Ma, Xiang Cui, Jian Zhang

**Affiliations:** 1 Department of Otolaryngology Head and Neck Surgery, Ningbo Medical Center Lihuili Hospital, Ningbo, Zhejiang, China; 2 Department of Blood Transfusion, Ningbo Medical Center Lihuili Hospital, Ningbo, Zhejiang, China

**Keywords:** isthmus, lymph node metastasis, nomogram, papillary thyroid microcarcinoma, prediction model

## Abstract

**Aim:**

To better predict the central lymph node metastasis (CLNM) of patients with isthmic papillary thyroid microcarcinoma (IPTMC) before surgery, we developed a new predictive nomogram based on clinical and ultrasound features and validate its reliability.

**Methods:**

Our study included 160 patients who were hospitalized from January 2016 to December 2024, underwent thyroidectomy with lymph node dissection, and were pathologically diagnosed with IPTMC. These patients were randomly divided into a training group of 112 cases and a validation group of 48 cases. Clinical and ultrasound characteristic data of the patients were collected. Univariate and multivariate logistic regression analyses were conducted on the training group to determine the independent risk factors for CLNM, and a nomogram was established based on these factors to predict the risk of CLNM in patients with IPTMC. The predictive performance of the nomogram was verified using the validation group.

**Results:**

Among the clinical and ultrasound features in the training cohort, we identified four independent risk factors for CLNM: age, tumor size, multifocality, and calcification. A predictive nomogram was developed based on the above four risk factors. The predictive nomogram showed excellent calibration in predicting CLNM, with an area under the curve (AUC) of 0.811 and a concordance index (C-index) of 0.783. The calibration curve of the nomogram was close to the ideal diagonal. In addition, decision curve analysis (DCA) proved that the model had significantly greater net benefits. The validation group verified the reliability of the prediction nomogram.

**Conclusions:**

The nomogram model developed in this study can effectively predict the risk of CLNM in patients with IPTMC before surgery and provide a reference for selecting surgical procedures.

## 1 Introduction

The thyroid gland, the largest endocrine organ in the human body, is divided into left and right lobes and located below the thyroid cartilage in a butterfly shape. The thin tissue band connecting the two lobes is called the isthmus, mainly located in front of the 2nd to 4th tracheal ring. In the past 40 years, the incidence of thyroid cancer has gradually increased worldwide (1). Papillary thyroid carcinoma is the most common type of thyroid cancer, accounting for about 85% of thyroid malignant tumors (2). Among them, the diameter <1 cm is called papillary thyroid microcarcinoma (PTMC), and it has the best prognosis of all thyroid cancers. Unilateral lobectomy and isthmus resection is feasible for PTMC confined to unilateral lobe, and the risk of postoperative recurrence is very low. Active surveillance can also be used for some low-risk patients (3). Nonetheless, occult lymph node metastasis in PTMC is still as high as 42.4%–50% (4, 5). Isthmic differentiated thyroid cancer accounts for about 1%–10% of all thyroid malignant tumors (6). Due to its special location, the cancer foci here are more likely to show capsular invasion and lymph node metastasis (6, 7). In the past, radical total thyroidectomy and prophylactic bilateral or ipsilateral central lymph node dissection were considered to be the most appropriate surgical methods for thyroid isthmus cancer (8). With the deepening of the understanding of thyroid cancer, the choice of surgical methods for thyroid cancer tends to be conservative (9). However, it still lacks guidelines for the surgical resection range of isthmic papillary thyroid microcarcinoma (IPTMC) and the range of prophylactic lymph node dissection. American Thyroid Association (ATA) management guidelines report that CLNM serves as an independent risk factor for recurrence of thyroid cancer (10).

Thyroidectomy combined with lymph node dissection is a common surgical procedure for PTMC patients with positive lymph nodes. Because of the relative indolent nature of PTMC and the risk of recurrent laryngeal nerve and parathyroid injury during the surgical procedure, it remains controversial whether to perform prophylactic central lymph node dissection in patients without detectable CLNM before surgery. Su and Li (11) suggested that prophylactic neck lymph node dissection could reduce the postoperative recurrence rate in patients with PTMC. However, Ywata de Carvalho et al. (12) suggested that it does not reduce the local recurrence rate, but increases the risk of nerve and parathyroid function damage. Due to the limitation of imaging technology, the preoperative detection rate of CLNM is low. The sensitivity of Contrast-Enhanced Ultrasound to detect LNM in patients with thyroid cancer was 33%–71.00% (13, 14). Considering the high metastasis rate of central lymph node and the complications after lymph node dissection, an effective and reliable preoperative tool capable of assessing lymph node metastasis is essential for optimizing surgical planning in patients with thyroid cancer.

Therefore, we developed a predictive nomogram based on clinical and ultrasound features to assess the risk of CLNM in patients with IPTMC.

We hope that our predictive nomogram will serve as a valuable reference for clinicians in developing personalized treatment strategies for patients with IPTMC.

## 2 Materials and methods

### 2.1 General information

Patients treated in our hospital from January 1, 2016 to December 31, 2024 with a diagnosis of “thyroid isthmus mass” and underwent surgical treatment. Inclusion criteria: ① postoperative pathological confirmation of IPTMC; ② patients who underwent their first thyroid surgery. Exclusion criteria: ① postoperative pathology indicated that the thyroid combined with other types of cancer, or combined with lateral lobe papillary carcinoma ≥1 cm; ② lost to follow-up or incomplete information; ③ did not receive prophylactic lymph node dissection; ④ CLNM was considered preoperatively.

### 2.2 Data collection

By utilizing electronic medical record systems and examination search systems, comprehensive collection of patients’ basic information can be achieved, including gender, age, preoperative thyroid ultrasound, surgical records, postoperative pathology reports, and more. The characteristics assessed by ultrasound examination included the number of tumors, multifocality, tumor size, calcification, margin, and echogenicity. Risk stratification was performed according to the Chinese TIRADS criteria, with nodules classified as category 4A or higher considered suspicious for malignancy (15). In this study, multifocality was defined as the presence of more than one nodule of category 4A or above, while tumor size referred to the maximum diameter of the suspected malignant nodule.

### 2.3 Statistical analysis

Data analyses were performed with SPSS (version 23.0) and R software (version 4.3.2). Categorical variables are expressed as numbers and percentages, while continuous variables are presented as means ± standard deviations or median (interquartile range [IQR]) depending on their distribution. For categorical data, Pearson's chi-square test or Fisher's exact test was utilized, while two-way analysis of variance was used for continuous variables. Patients were randomly divided into training group (70%) and validation group (30%). Univariate logistic regression analysis was performed on the clinical characteristics and ultrasound characteristics of patients in the training group, and the significant factors (*P* < 0.1) were identified and included as independent variables, followed by multivariate binary logistic regression analysis to identify the independent risk factors for CLNM (*P* < 0.05). Then, the rms/pROC package in R software was used to construct a predictive nomogram based on the results of the multivariate logistic regression. The prediction accuracy and consistency of the model were assessed using the ROC curve, area under the ROC curve (AUC), and calibration curve. Additionally, decision curve analysis (DCA) was employed to evaluate the net benefit of the prediction model for patients. The validation group was used to verify the predictive performance of the nomogram.

This study was approved by the Ethics Committee, which waived the requirement for informed consent due to retrospective nature of the study.

## 3 Results

### 3.1 Patient information

The data of 160 patients with IPTMC were collected in this study. The study included 41 (25.6%) men and 119 (74.4%) women, with a median age of 47 years (IQR: 37–55; range 24–71). The median tumor size was 5.5 mm (IQR: 4.0–7.0; range 2–9). CLNM was observed in 62 patients (38.8%), and the multifocality was found in 40 patients (25.0%). All patients were randomly divided into a training group (*n* = 112) and a validation group of 48 (*n* = 48). Table 1 shows the information for each group.

**Table 1:** Comparisons between the training group and validation group.

Factor	Training group (112)		Validation group (48)		*P*
	CLNM+ (%)	CLNM− (%)	CLNM+ (%)	CLNM− (%)	
Gender					0.182
male	14 (12.5%)	12 (10.7%)	9 (18.8%)	7 (14.6%)	
female	26 (23.2%)	60 (53.6%)	13 (27.1%)	19 (39.6%)	
Age (Year)					0.607
>55	9 (8.0%)	21 (18.8%)	2 (4.2%)	9 (18.8%)	
<55	31 (26.7%)	51 (45.5%)	20 (41.7%)	17 (35.4%)	
Tumor size (mm)	7.0 (5.0, 8.0)	5.0 (4.0, 6.0)	7.0 (5.0, 8.0)	5.5 (4.0, 7.0)	0.440
Multifocality	16 (14.3%)	12 (10.7%)	7 (14.6%)	5 (10.4%)	1.000
Calcification	18 (16.1%)	14 (12.5%)	10 (20.83%)	5(10.4%)	0.733

Tumor size data are median (Q1, Q3).

### 3.2 Risk factors

Univariate logistic regression analysis conducted on the training group revealed that age (*P* = 0.076), tumor size (*P* = 0.01), multifocality (*P* = 0.008), calcification (*P* = 0.005), and gender (*P* = 0.031) were risk factors for CLNM of IPTMC. Multivariate binary logistic regression analysis indicated that age (*P* = 0.025), tumor size (*P* = 0.002), multifocality (*P* = 0.003) and calcification (*P* = 0.008) were independent risk factors for CLNM in IPTMC. Table 2 presents the specific analysis data. Univariate and multivariate binary logistic regression analysis conducted on the patients without multifocality (*n* = 120) both indicated consistent results (Table 3).

**Table 2:** Univariate and multivariate logistic regression analysis of risk factors for CLNM in training group.

Risk factors	Univariate regression analysis			multivariate regression analysis		
	OR	95% CI	*P*	OR	95% CI	*P*
Age (year)	0.968	0.934–1.003	**0.076**	0.949	0.907–0.993	**0.025**
Tumer size (mm)	1.410	1.142–1.740	**0.01**	1.498	1.164–1.929	**0.002**
Multifocality						
Positive	3.333	1.375–8.082	**0** **.** **008**	5.307	1.736–16.221	**0.003**
Negative	1 (reference)			1 (reference)		
Calcification						
Positive	3.390	1.444–7.959	**0** **.** **005**	4.308	1.477–12.570	**0.008**
Negative	1 (reference)			1 (reference)		
Gender						
Female	0.371	0.151–0.912	**0** **.** **031**	0.355	0.125–1.010	0.052
Male	1 (reference)			1 (reference)		
Margin						
Well-defined	1.40	0.611–3.209	0.427			
Ill-defined	1 (reference)					
Echogenicity						
Echogenic foci	2.032	0.733–5.631	0.173			
Hypoechoic	1 (reference)					

Bold values highlight statistically significant differences for quick identification.

**Table 3:** Univariate and multivariate logistic regression analysis of risk factors for CLNM in patients without multifocality (*n* = 119).

Risk factors	Univariate regression analysis			multivariate regression analysis		
	OR	95% CI	*P*	OR	95% CI	*P*
Age (year)	0.940	0.905–0.976	**0** **.** **001**	0.919	0.879–0.961	**<0** **.** **001**
Tumer size (mm)	1.510	1.206–1.892	**<0** **.** **001**	1.593	1.236–2.053	**<0** **.** **001**
Calcification						
Positive	2.539	1.090–5.916	**0** **.** **031**	4.822	1.663–13.979	**0** **.** **004**
Negative	1 (reference)	1 (reference)				
Gender						
Female	0.571	0.245–1.333	0.195			
Male	1 (reference)					
Margin						
Well-defined	1.906	0.840–4.325	0.123			
Ill-defined	1 (reference)					
Echogenicity						
Echogenic foci	2.194	0.841–5.728	0.108			
Hypoechoic	1 (reference)					

Bold values highlight statistically significant differences for quick identification.

### 3.3 Construct the predictive nomogram

**Figure 1:**
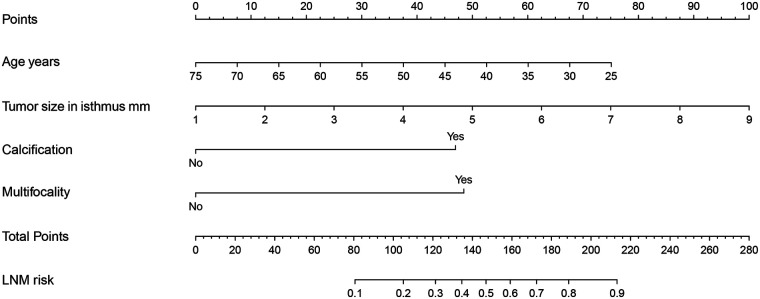
Risk prediction nomogram of CLNM in patients with IPTMC.

A predictive nomogram was constructed based on the four identified independent risk factors (C-index = 0.783) (Figure 1). The higher total score indicates the higher risk of CLNM in patients with IPTMC. When utilizing this predictive nomogram in an individual patient, information corresponding to the four risk factors (axes 2–5) should be represented as point values projected onto the first axis. Subsequently, the sum of these four points should be plotted on axis 6. Then, the point corresponding to the total score on the axis 7 represents the probability of lymph node metastasis in the patients with IPTMC.

### 3.4 Accuracy and net benefit of the predictive nomogram

**Figure 2:**
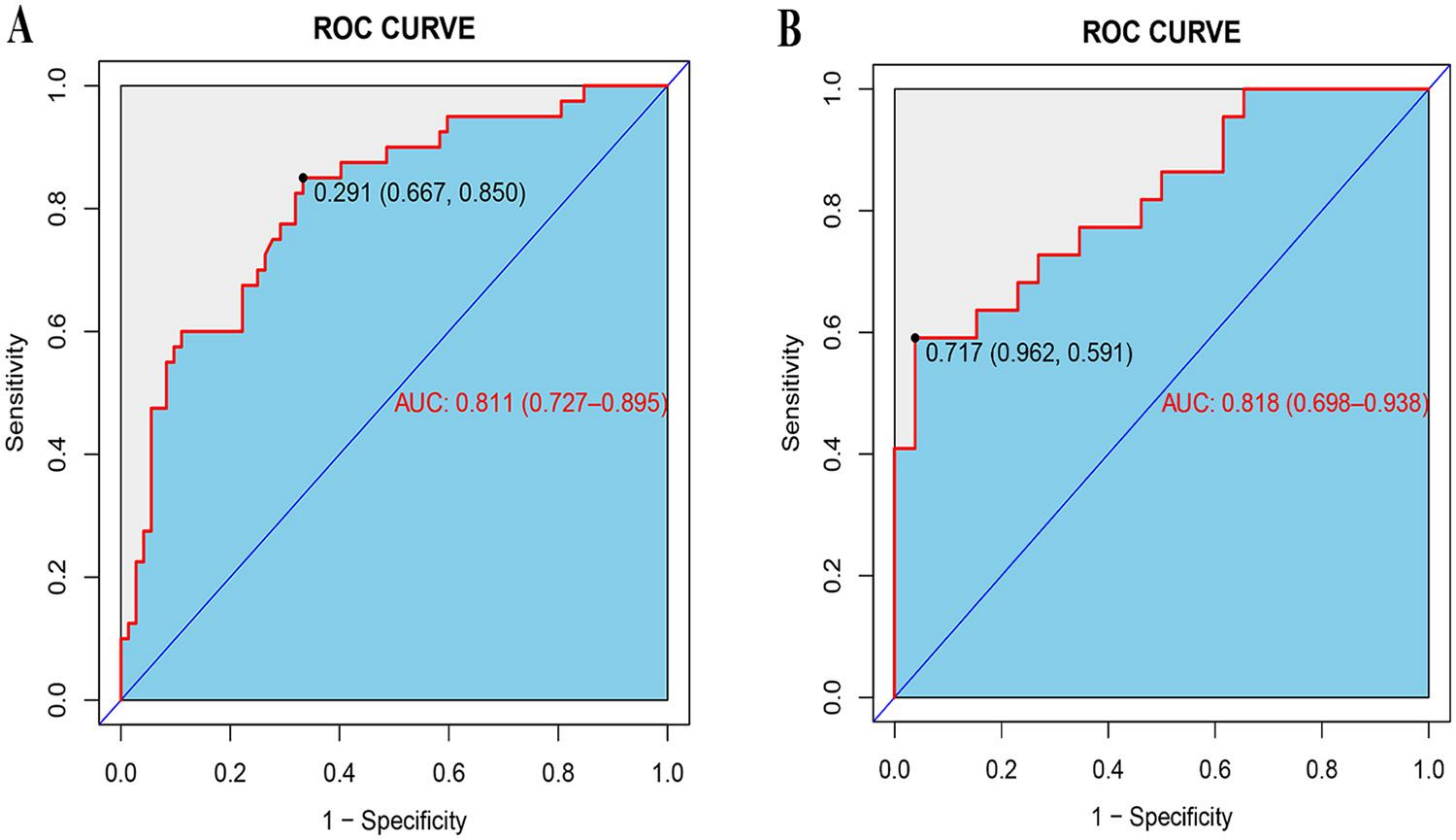
ROC curves. **(A)** Training group. **(B)** Validation group.

**Figure 3:**
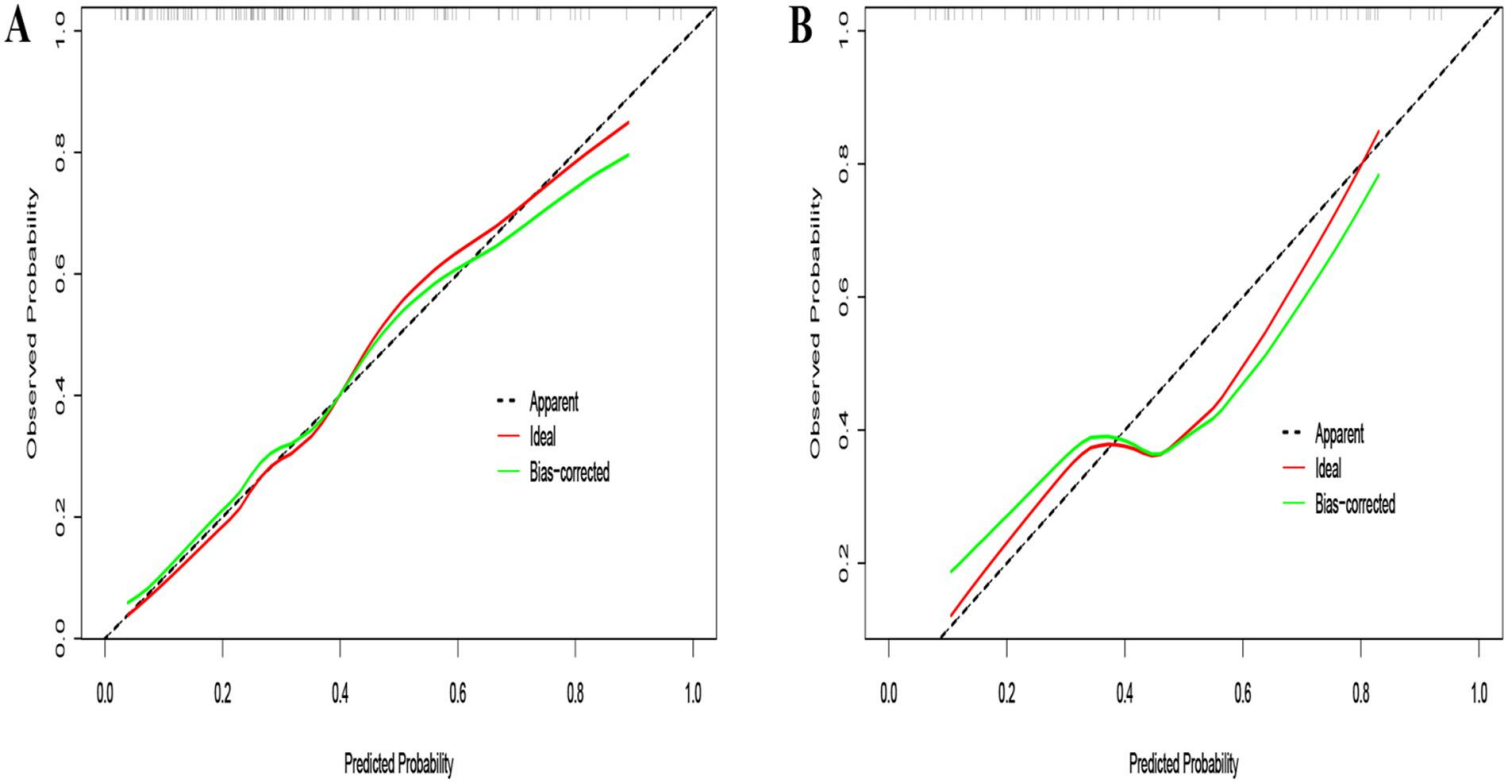
Calibration curve. **(A)** Training group. **(B)** Validation group.

**Figure 4:**
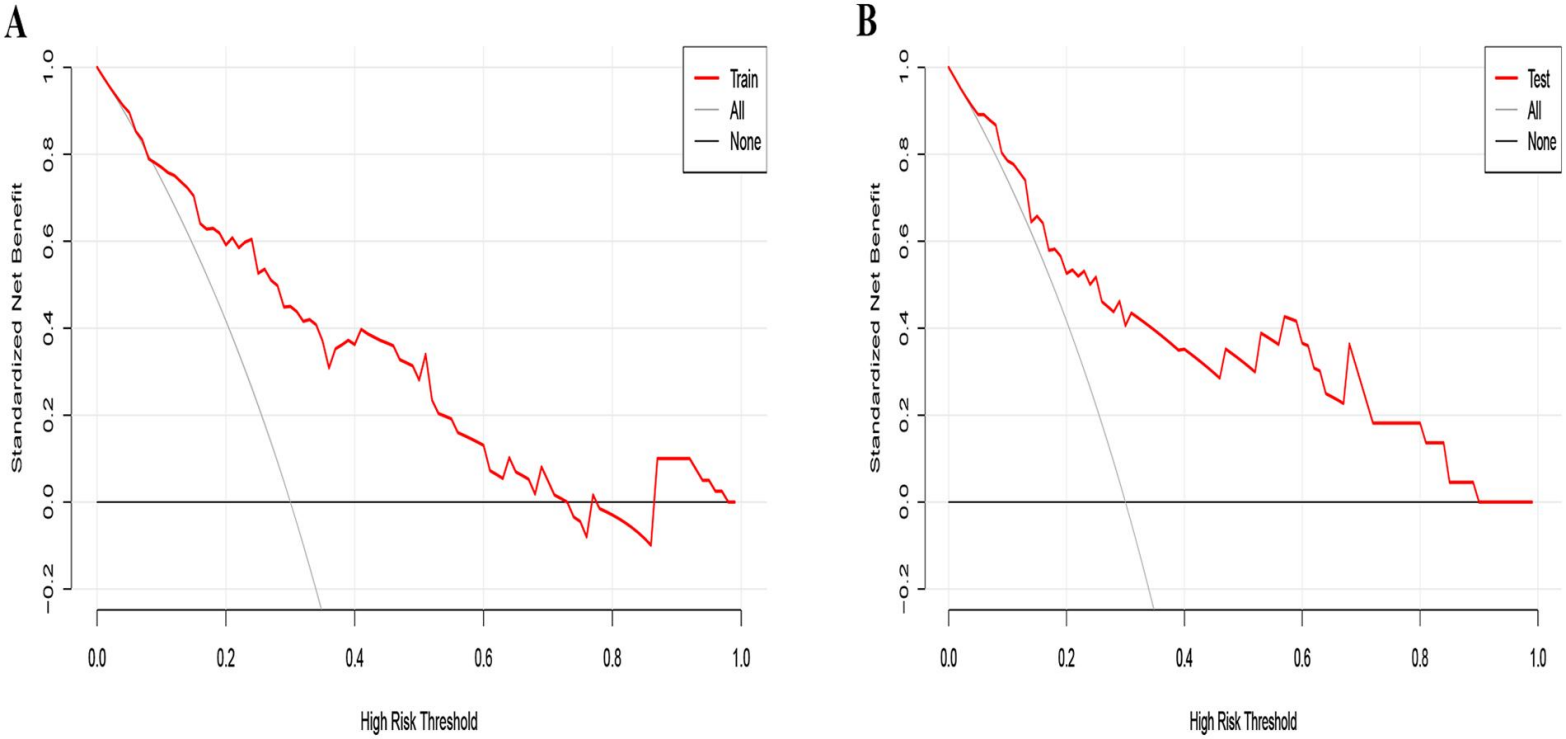
Decision curve analysis in prediction of CLNM in IPTMC. **(A)** Training group. **(B)** Validation group.

The ROC curve was drawn from the data of the training group and the AUC was 0.811 (Figure 2A), with the calibration curve closely aligning with the ideal diagonal (Figure 3A). Additionally, DCA revealed that the nomogram provided a substantially higher net benefit (Figure 4A). Furthermore, the nomogram was tested using the validation cohort of 48 patients from our hospital, yielding an AUC of 0.818 (Figure 2B), which reflects the nomogram's good accuracy. The calibration curve for the validation group also exhibited strong alignment with the ideal diagonal (Figure 3B). In addition, DCA demonstrated a substantial net benefit for the nomogram in the validation group (Figure 4B). These findings suggest that the nomogram has considerable value for practical clinical use.

## 4 Discussion

Patients with PTMC often have no obvious clinical symptoms in the early stage and are usually diagnosed during physical examinations. PTMC is characterized by low degree of malignancy, effective treatment, and good prognosis. As a relatively special region of the thyroid gland, the cancerous lesions in the isthmus are more likely to have CLNM. Preoperative imaging examinations have limited sensitivity in detecting lymph node metastasis currently (16). Therefore, it is very necessary to establish an effective and reliable prediction nomogram to predict CLNM in IPTMC.

Studies have showed that male gender, multifocality, tumor size >5 mm, extrathyroidal extension (ETE), ultrasound imaging-suggested tumor boundaries and bilaterality are reliable clinical predictors of CLNM in patients with C_N0_ PTMC (17–20). In recent years, there have been numerous reports on predictive nomograms for CLNM in PTMC. Zhao et al. (21) analyzed the isolated thyroid cancer in the isthmus and identified three independent risk factors of gender, age and tumor size, and established nomograms to predict the risk of ipsilateral and contralateral CLNM. The area under the ROC curve were both 0.779, and the C-index was 0.756 and 0.753. Wang et al. (22) found that male gender, younger age, smaller tumor diameter, ETE, presence of microcalcification, multifocality and absence of Hashimoto's thyroiditis were risk factors for CLNM of PTMC, and AUC and C-index of their predictive model were both 0.755. Ye et al. (23) found that sex, tumor size and ETE were risk factors for CLNM of PTMC and the AUC of their predictive model was 0.684. The AUC of our predictive nomogram was 0.811, and the C-index was 0.783, indicating that our nomogram may have better predictive value in predicting CLNM of patient with IPTMC.

In previous studies, gender has been considered as an independent risk factor. Through univariate and multivariate logistic regression analysis, gender was identified as a risk factor (*P* = 0.031) but was not found to be an independent risk factor. (*P* = 0.052). This may be related to the small sample size, but it does not affect the application value of our predictive model. Consistent results were obtained from univariate and multivariate logistic regression analyses across both the training group and the non-multifocality patient group, identifying a congruent set of independent risk factors other than multifocality. We did not exclude patients with PTMC in the lateral lobe, which may bias our results but is more clinically relevant because of the high incidence of multifocality. Previous studies reported that the incidence of multifocal carcinoma of PTMC was 28.1% (24), which was consistent with our study. Among the risk factors, multifocality had the largest OR value, and the risk of CLNM in patients with multifocal carcinoma was about 5.3 times higher than that with unifocal carcinoma. Therefore, these patients cannot be ignored.

In this study, the incidence of CLNM was 38.8%, slightly lower than the 44% reported in previous studies (25). However, the presence of occult CLNM cannot be overlooked, as it significantly influences the choice of surgical approach. Although prophylactic central lymph node dissection may reduce the risk of lymph node metastasis, it could also lead to a higher rate of complications following thyroid surgery. With a deeper understanding of thyroid cancer, the selection of surgical methods for thyroid cancer tends to be more conservative. In recent years, thyroid radiofrequency ablation technology has been applied in clinical practice. This technique has garnered attention due to its advantages of minimal invasiveness, fewer complications, rapid recovery, and improved quality of life for patients (26, 27). Nonetheless, this method may overlook occult CLNM, and its long-term efficacy requires further clinical follow-up studies for validation. Therefore, it is particularly important to conduct a rigorous preoperative assessment of surgical indications for patients opting for this type of surgical procedure. Moreover, the ability to more accurately identify and predict lymph node metastasis preoperatively is crucial for formulating appropriate surgical strategies and enhancing patient prognosis.

Our study developed a predictive tool based on preoperative ultrasound features; however, it is essential to acknowledge the limitations of ultrasound in accurately determining the presence of malignant thyroid nodules. The “multifocality” identified by ultrasound may not fully align with the pathologically confirmed multifocality from histopathological examination. This discrepancy represents a fundamental constraint for any preoperative model relying on imaging data. In our study, final pathological examination confirmed multifocal cancer in 41 cases. Ultrasound detected suspected multifocality in 40 cases, of which 33 were later pathologically confirmed, resulting in a sensitivity of 80.5% and a specificity of 94.1%. It should be noted that these performance metrics are inherently dependent on the quality of the ultrasound equipment and the expertise of the sonographer. Therefore, the risk stratification provided by our tool should be interpreted as the optimal assessment achievable with the currently available preoperative information. It quantifies the malignancy risk associated with lesions that can be observed and measured prior to surgery, thereby aiding clinical decision-making.

Our study presents an effective predictive model. ROC curve analysis identified a clear cutoff value of 0.291 for the risk of lymph node metastasis, corresponding to a total nomogram score of 120.0 points. When the total score exceeds 120.0, the risk of lymph node metastasis increases significantly. In clinical practice, thyroid ultrasound is used to evaluate nodules, assessing features such as size, multifocality, calcification, and TI-RADS grade. In combination with clinical features, we recommend performing prophylactic lymph node dissection when the total score exceeds 120.0.

Our study still has some limitations. First, the sample size of the study was not large enough. Therefore, it is essential to expand the sample size to enhance the robustness and accuracy of the nomogram and conduct external validation in the future. Second, the performance of the nomogram may be partially influenced by variations in instruments and differences in operator expertise. Thirdly, the nomogram model was developed mainly for patients with papillary thyroid microcarcinoma located in the isthmus, and its applicability to more patients with other types of thyroid cancer needs to be further verified.

In conclusion, the nomogram we developed demonstrates good predictive capability for assessing the risk of CLNM in patients with IPTMC. It serves as a valuable tool to assist clinicians in developing individualized treatment plans.

## Data Availability

The original contributions presented in the study are included in the article/Supplementary Material, further inquiries can be directed to the corresponding author.
